# Augmented Reality Guided Needle Biopsy of Soft Tissue: A Pilot Study

**DOI:** 10.3389/frobt.2020.00072

**Published:** 2020-06-16

**Authors:** David Asgar-Deen, Jay Carriere, Ericka Wiebe, Lashan Peiris, Aalo Duha, Mahdi Tavakoli

**Affiliations:** ^1^Electrical and Computer Engineering, University of Alberta, Edmonton, AB, Canada; ^2^Oncology, Medicine and Dentistry, University of Alberta, Edmonton, AB, Canada; ^3^Surgery, Medicine and Dentistry, University of Alberta, Edmonton, AB, Canada; ^4^Radiology and Diagnostic Imaging, University of Alberta, Edmonton, AB, Canada

**Keywords:** biopsy, augmented reality (AR), needle guidance, minimally invasive surgery (MIS), oncology

## Abstract

Percutaneous biopsies are popular for extracting suspicious tissue formations (primarily for cancer diagnosis purposes) due to the: relatively low cost, minimal invasiveness, quick procedure times, and low risk for the patient. Despite the advantages provided by percutaneous biopsies, poor needle and tumor visualization is a problem that can result in the clinicians classifying the tumor as benign when it was malignant (false negative). The system developed by the authors aims to address the concern of poor needle and tumor visualization through two virtualization setups. This system is designed to track and visualize the needle and tumor in three-dimensional space using an electromagnetic tracking system. User trials were conducted in which the 10 participants, who were not medically trained, performed a total of 6 tests, each guiding the biopsy needle to the desired location. The users guided the biopsy needle to the desired point on an artificial spherical tumor (diameters of 30, 20, and 10 mm) using the 3D augmented reality (AR) overlay for three trials and a projection on a second monitor (TV) for the other three trials. From the randomized trials, it was found that the participants were able to guide the needle tip 6.5 ± 3.3 mm away from the desired position with an angle deviation of 1.96 ± 1.10° in the AR trials, compared to values of 4.5 ± 2.3 mm and 2.70 ± 1.67° in the TV trials. The results indicate that for simple stationary surgical procedures, an AR display is non-inferior a TV display.

## 1. Introduction

Percutaneous biopsies are commonly performed by radiologists to extract tissue samples from a patient to aid in making diagnoses. This procedure is performed by inserting a biopsy needle (see **Figure 2B**) into the skin and guiding it to the area of interest. Once correctly positioned, a core sample can is extracted by cutting a piece of the soft tissue. Often, an automated or semi-automated device is used to cut and store the soft tissue in the notch of the inner needle. The needle is often guided to the area of interest using a form of imaging including ultrasound (US), magnetic resonance imaging (MRI), and mammograms (for stereotactic guidance) (Liberman, 2000).

Core needle biopsies are the main alternative to surgical biopsies as they are less expensive, less invasive, result in minimal scarring, can be performed quickly, and are lower risk for the patient (Parker et al., 1994; Liberman, 2000). Despite the benefits of percutaneous biopsies, the rate of false negatives for breast biopsies was found to be between 2.2 and 2.5% (Jackman and Marzoni, 1997; Boba et al., 2011). The most common reasons for the false negatives include using the wrong imaging method during the biopsy procedure (Boba et al., 2011) and having poor visualization of the lesion/needle during the operation (Youk et al., 2007).

**Figure 1:**
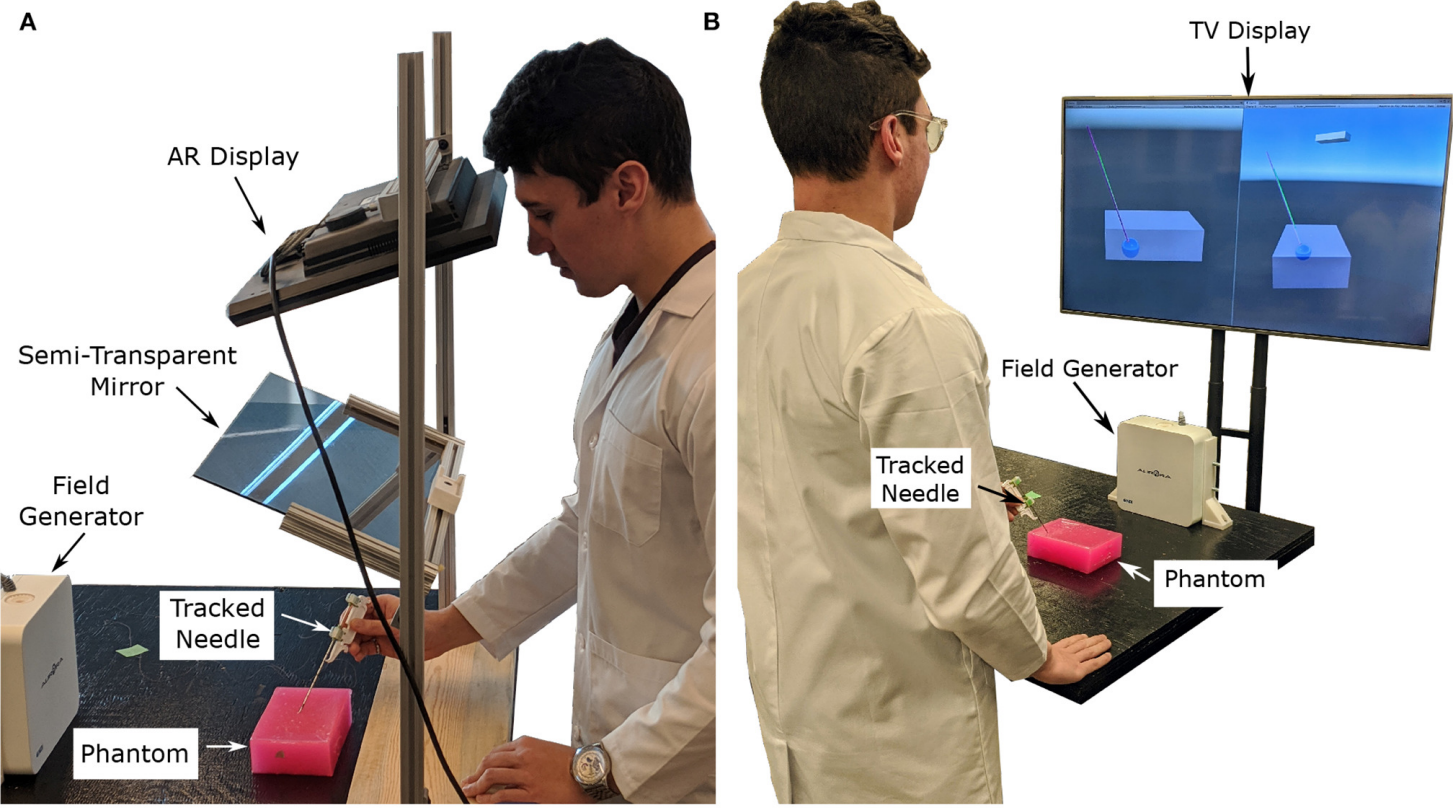
Images showing the two display modalities used for the experiments. For both display modalities, the tracked needle position, tumor phantom model, and desired needle trajectory are shown to the user. An Aurora V2 planar field generator is used to track two 6-DOF sensors attached to a biopsy needle, and one 6-DOF sensor affixed to the side of the phantom. **(A)** The semi-transparent mirror AR display used for the experiments. The AR setup includes a Kinect V2 system (out of frame) for head tracking. **(B)** The TV display used for the experiments showing information from a forward and side profile.

**Figure 2:**
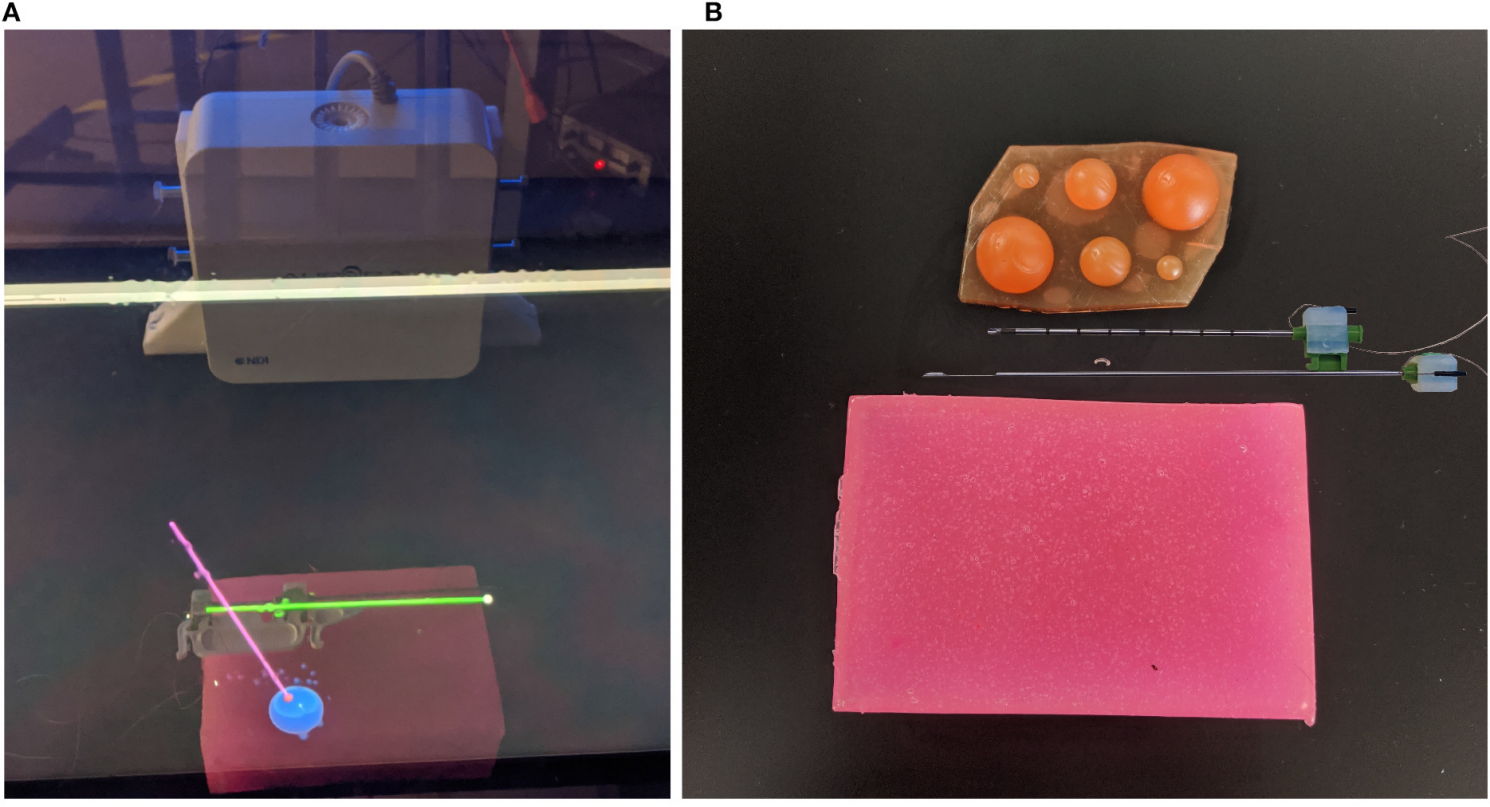
Images of AR display, phantom tissue, and artificial tumors. In **(A)** the green cylinder represents the tracked needle (with a white spherical tip), the blue sphere is the tracked tumor, and the pink cylinder represents the ideal trajectory (with a red sphere on the end for the needle tip desired position). In **(B)** the top needle (outer needle) is hollow and is used to guillotine the soft tissue sample. The bottom needle (inner needle) contains the notch measuring 18 mm in length, which will hold the soft tissue sample. **(A)** This image shows the AR display the user sees during the trials. **(B)** This image shows the phantom used for the experiments (pink brick at the bottom of the image), the disassembled sensorized 14 gauge core biopsy needle, and the artificial tumors embedded within the phantom.

The authors have attempted to address the concern of poor visualization by creating two distinct setups. The first was an augmented reality setup with head tracking, which allows the operator to visualize both the tumor and the needle through an opaque phantom (see Figures 1A, 2B). The second setup included both front and side views of the needle operation are displayed on a secondary TV screen away from the phantom (see Figure 1B). Augmented reality has been used for other for medical procedures (Birkfellner et al., 2002; Harders et al., 2007; Fong et al., 2019; Ocampo and Tavakoli, 2019), further adding to the validity of implementing this technology into biopsy procedures.

In this experiment, users with no prior experience with biopsying were asked to localize the needle to an ideal end position using both the augmented reality setup and TV virtualization setup (see Figures 1A,B, respectively). The users attempted the localization procedure for each size of the tumor (30, 20, and 10 mm) using both setups for a total of 6 trials. Specific needle trajectories were given to the participants increasing the difficulty of the task and highlighting the benefits of the system. Information on the speed (time to perform each trial), accuracy (Euclidean distance and angle offset of ideal and actual needle tips), and users' subjective experience were collected throughout the trials.

In this paper, the results of needle localization using two different virtualization systems were compared to see if AR guided biopsy has comparable results to a more traditional secondary display setup. Additionally, this paper also shows how inexperienced individuals can obtain sub-centimeter and ±5° needle placement accuracy using the proposed systems making a strong case to bring new visualization technologies into the operating room. This pilot study will help future researchers determine what sample sizes they should choose for their tests, and what factors they should consider when developing their trials. This research was approved by the University of Alberta Research Ethics board under approval number Pro00070096.

Information relating to the benefits of percutaneous biopsies, current visualization methods proposed and used for biopsying, and the need for better visualization methods are covered in section 2. The rationale behind the phantom parameters and other experimental setup design choices are covered in section 4. In section 3, the experimental procedure is described to the reader, along with other technical information. The data obtained from the trials, along with an analysis of said data, can be found in section 6. Lastly, an interpretation of the results comes in section 6 and a discussion on how to improve the results in the future are in section 7.

## 2. Background and Motivations

### 2.1. Percutaneous Biopsies

Percutaneous biopsies are an essential surgical procedure that allow pathologists to examine abnormal tissue within the body. Often these abnormal tissue formations are found using several types of imaging modalities, including ultrasound, x-ray, MRI, single-photon emission computed tomography (SPECT), positron emission tomography (PET), and optical imaging (Frangioni, 2008).

In a retrospective analysis of 988 biopsies performed between March 2006 and February 2008, Boba et al. (2011) found that 22 cases (2.2%) resulted in a false negative finding. The primary reasons for the false negatives found in this study include using the wrong imaging method during the biopsy procedure, choosing the wrong biopsy system, improper monitoring of the needle location, and poor visualization of the lesion or needle (Boba et al., 2011). In terms of false negatives caused by wrong imaging methods, Marek Boba et al. found that performing a biopsy using US instead of stereotactic guidance provided the physician with better control of the sampling process, real-time guidance, direct visualization of the needle, and faster procedure times. In a separate analysis performed by Liberman et al., it was found that out of 144 core biopsies performed, five false negatives (3.5%) were caused by inaccurate needle placement (Liberman et al., 1997). A core biopsy involves extracting a small piece of soft tissue from a larger system similar to the one in Figure 2B. The rate of false negatives typically decreased as the radiologist became more experienced (Brenner et al., 1996; Liberman et al., 2001).

From the above analysis, it is clear that there is a need for needle/lesion visualization for biopsy procedures. Furthermore, it would be beneficial to see whether there is a clear advantage to relaying this information superimposed over the patients' skin (using an AR setup similar to Figure 1A) or if a separate display (similar to Figure 1B), works well-enough.

### 2.2. Imaging Modalities

There are several different imaging modalities available for performing biopsies. One of the most common imaging modalities is ultrasound, as it is relatively cheap (compared to MRI, PET, and SPECT), readily available, safe for the patient and physician, allows for real-time tracking, and offers excellent contrast between soft tissue. Most biopsy setups use the ultrasound scanner to visualize the tumor and to track the needle.

Visualization of the needle can be difficult using ultrasound guidance as only a cross-section of the needle can be seen in a typical two-dimensional ultrasound image. There are two main ways to capture the needle within an image: normal-plane (or transverse-plane) imaging or longitudinal-plane (or sagittal-plane) imaging. A longitudinal-plane image can show valuable information about the position of the needle but requires a steady grip on the ultrasound probe to ensure it stays within frame, especially if the needle deflects out of the longitudinal plane. Obtaining a normal-plane image is easier, but determining where the needle tip is located is more complicated.

Three-dimensional images can be created from a series of two-dimensional ultrasound slices using online or offline reconstruction techniques (Huang and Zheng, 2008; Huang and Zeng, 2017). These volumes can also be obtained through MRI or CT scans. As these volume reconstructions are often done preoperatively, these images must be registered to the intraoperative scans using different types of rigid and non-rigid registration techniques (Estépar et al., 2009; Gillies et al., 2017; De Silva et al., 2018). As this study focuses on the effects of different visualization setups, it will be assumed that a perfect model of the tumor is available and correctly registered to the phantom. The previously mentioned reconstruction techniques are included to show that this imaging method is viable in a real-world scenario.

### 2.3. Comparable Systems

Comparing these results with other similar research, a robot-assisted system proposed by Kettenbach et al. performed a similar trial through robotic-assisted biopsy in which the insertion depth ranged from 10 to 70 mm (Kettenbach et al., 2005). This system was able to position a guide for the biopsy needle to slide through for a manual biopsy. However, from the illustrations in the provided figure, it appears the robot was only able to rotate about one axis. The systems positioning accuracy along the x-axis was 1.2 ± 0.8 and 1.4 ± 0.9 mm along the z-axis with a procedure time of 2.6 ± 1-min. No y-axis deviations or angle information was provided.

## 3. Experimental Outline

### 3.1. Experimental Procedure

**Figure 3:**
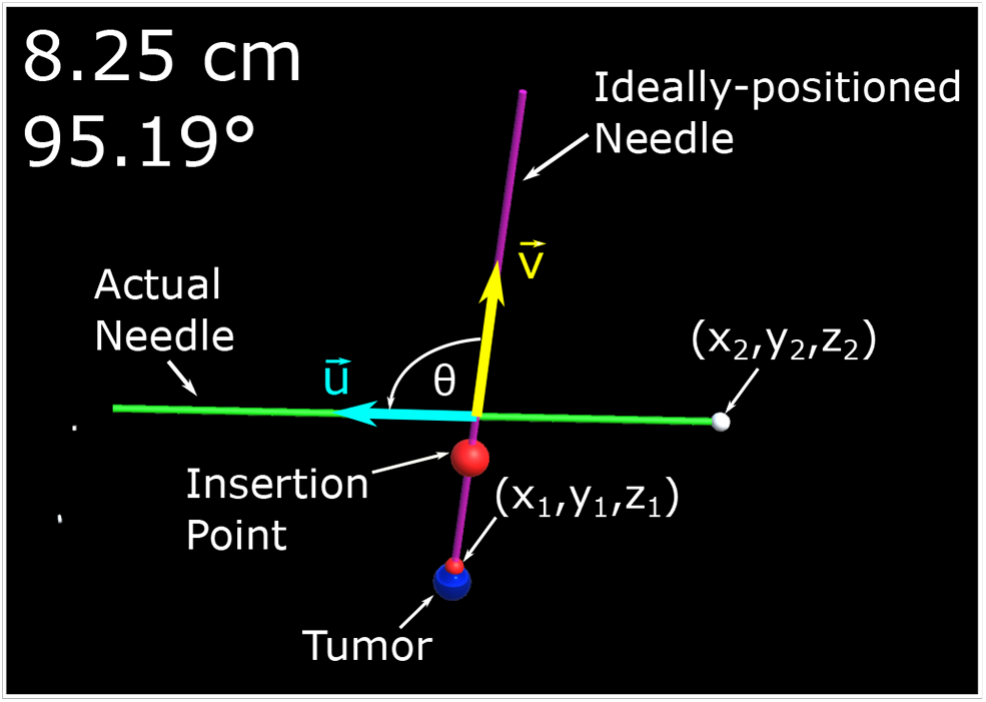
This image is a screenshot of what is displayed on the monitor of the AR setup with manual annotations. In the top left hand corner are two numbers. The number in the first row represents the Euclidean distance of the desired needle tip position (*x*_1_, *y*_1_, *z*_1_) compared to the actual tracked needle tip position (*x*_2_, *y*_2_, *z*_2_). The number in the second row represents the angle difference of the ideal and actual needle represented in degrees. The angle between the two needles, θ, is the angle between $\vec{u}$ (red arrow vector) and $\vec{v}$ (blue arrow vector) as defined by (2).

The experiment begins with a coin flip to determine which setup (i.e., TV setup and AR setup) will be used first. If the coin flip is heads then the participant will begin with the AR setup, if tails the second screen visualization will be used. The participant will be standing at the edge of the table ~300–350 mm away from the phantom. The person running the experiment will then make one of the 30 mm tumors visible to the participant along with a desired trajectory. The participant is given a 1-min window to practice using the visualization setup before the tracked trials are started. Once the practice trial is completed, the angle for the ideal needle trajectory is changed and the participant is instructed to guide the needle to the end location of a displayed trajectory by attempting to get the two displayed numbers (Euclidean distance and angle offset) as close to zero as possible. It should be noted that participants were told not to worry about their procedure time as their focus was to decrease the two displayed errors as low as possible. The equation determining the Euclidean distance, δ*D*, of the two points can be found in (1) where (*x*_1_, *y*_1_, *z*_1_) represent the ideal end position of the needle tip and (*x*_2_, *y*_2_, *z*_2_) represents the needle tip's actual position all in the Unity frame. The ideal end position is positioned at the surface of the artificial tumor. Equation (2) shows how the angle, θ, between the two vectors, that lie along the long axis of the actual and ideal needle ($\vec{u}$ and $\vec{v}$, respectively) is calculated. Illustrations of these variables can be found in Figure 3.

(1) $$\begin{array}{l} \Delta D=\sqrt{{\left(x_{1}-x_{2}\right)}^{2}+{\left(y_{1}-y_{2}\right)}^{2}+{\left(z_{1}-z_{2}\right)}^{2}} \end{array}$$

(2) $$\begin{array}{l} \theta =arccos\left(\frac{\vec{u}\cdot \vec{v}}{\vec{\left||u|\right|}\cdot \vec{\left||v|\right|}}\right) \end{array}$$

Once the participant is ready to start the trial, the experimenter will begin logging the data after instructing the participant to begin the procedure. The logging ended when the participant felt that they had reached the desired end point. The time at which the participant felt they reached the end destination as best as they could was recorded. The data logged includes the time stamp of when the data was captured, the Euclidean distance of the needle desired and actual needle tips (*D*), the angle between the ideal and actual needle (θ), and the timestamp of when the final destination was reached.

The same procedure (minus the 1-min training period) is performed for the 20 mm tumor and then the 10 mm tumor. Once three needle localization's have been performed using one type of visualization method, chosen through a coin flip, the participant performs the procedures using the other method. Once again the participants will get the 1-min practice trial at the beginning to get acquainted with the new visualization display. It should be noted that the angle of insertion is varied after each trial to introduce a level of variability that could be seen in an operating room and to avoid learning carry-over. Information relating to the ideal needle trajectory angles can be found in section 4.1.

Once all surgical procedures were performed, the participant was then instructed to fill out a questionnaire rating their subjective experience with the system. The wording and results of the questionnaire can be found in section 6.4. It should be noted that the volunteers recruited to perform these tasks had no previous knowledge in performing surgical procedures, and are considered inexperienced at performing biopsies.

### 3.2. Visualization Displays

This experiment was split into two distinct set of trials. The two trials included an AR setup and a visualization on a second screen (TV setup). For both setups, the phantom was placed 300–350 mm away from the edge of the table. The device used to track the needle and tumor locations is the Aurora V2 system which includes a magnetic field generator and three 6-DOF electromagnetic trackers. It should be noted that a rigid transformation was found from the phantom tracker to the center of each tumor, and the tracking of the tumor is not the focus of the paper. The trackers are able to record the *x, y, z* positions with 0.7 mm root mean square (RMS) accuracy and rotation of the device (roll, pitch, yaw) with 1.3° RMS accuracy (Lugez et al., 2015). Each participant is instructed to perform a biopsy on all three tumor sizes (30, 20, and 10 mm) from largest to smallest using each visualization system. To ensure learning does not bias the results the participants were randomly chosen to start with either the AR system or the visualization on the second screen using a coin flip. The trial each participant started with can be seen in the second column of Table 1. It should be noted that the last column of both tables are exactly the same, and are replicated for easier reading.

**Table 1:** Quantitative results.

		**AR display**			**TV display**		
**Participant number**	**Initial trial**	**Insertion angle deviation (^**°**^)**	**Euclidean distance offset (mm)**	**Procedure time (s)**	**Insertion angle deviation (^**°**^)**	**Euclidean distance offset (mm)**	**Procedure time (s)**
1	AR	2.47 ± 0.56	12.71 ± 10.76	117.7 ± 30.7	4.41 ± 0.62	8.04 ± 1.38	67.9 ± 20.8
2	TV	0.89 ± 0.49	6.74 ± 3.83	70.9 ± 27.7	2.79 ± 2.16	4.93 ± 4.28	55.1 ± 39.8
3	TV	1.99 ± 0.52	8.59 ± 1.68	138.0 ± 135.8	1.47 ± 1.09	10.76 ± 11.55	69.9 ± 18.6
4	AR	4.60 ± 4.77	7.93 ± 4.25	170.4 ± 95.2	3.12 ± 3.03	5.93 ± 1.98	47.0 ± 16.6
5	TV	2.26 ± 0.37	6.41 ± 3.58	32.6 ± 3.6	2.75 ± 0.73	2.92 ± 1.44	31.5 ± 6.4
6	AR	2.18 ± 2.54	11.04 ± 10.43	40.9 ± 7.8	1.68 ± 1.14	2.59 ± 1.16	95.8 ± 43.5
7	AR	3.12 ± 2.49	8.29 ± 6.53	47.5 ± 4.5	2.55 ± 0.97	8.23 ± 1.45	53.3 ± 13.2
8	TV	4.67 ± 2.57	7.72 ± 2.90	49.0 ± 9.9	3.21 ± 2.65	3.92 ± 1.58	99.4 ± 34.6
9	TV	1.87 ± 1.41	8.19 ± 3.57	109.2 ± 26.0	2.29 ± 1.28	3.09 ± 0.92	105.6 ± 28.9
10	AR	1.47 ± 1.27	3.29 ± 2.62	67.3 ± 18.7	1.08 ± 1.01	4.71 ± 1.38	64.2 ± 0.5

In the AR setup, a piece of semi-transparent mirror is placed between the participant and the phantom. A monitor mounted above the mirror projects an image of the needle and tumor over the physical system (see Figure 1A). To ensure the image moves with the motion of the participants head, a head tracking algorithm was implemented using a Kinect V2 system. The Kinect V2 was positioned off to the side of the AR setup, where the physical position of the Kinect matches the position indicated in the model of the setup show in **Figure 5B**. Using a head tracking algorithm along with some modified camera projection matrix equations, the image appears overlaid directly over the physical system. This setup was chosen over a head-mounted AR system as historically head-mounted displays have not offered a suitable field of view for surgical applications (Keller and Colucci, 1998).

The setup used for the visualization on the second screen (TV setup) includes a similar visualization style as the AR setup except that the models of the needle and tumor are projected on a screen away from the physical system. Two different 2-D perspectives are given to the user in order to gain necessary spatial data. Both of these methods use the Plus Toolkit (Lasso et al., 2014) to stream the position data from the NDI tracking system to Unity. Unity is a powerful game engine that allows developers to create 3-D environments efficiently and effectively.

The two systems were created using the same environment (Unity Engine), trackers, and graphics to ensure comparisons between the two imaging modalities were fair. One key motivation behind creating two systems which differed primarily in their presentation of the visual data was to determine whether an AR setup would provide a benefit to physicians, or at least prove to be non-inferior component to the TV setup-based virtual reality environment. In section 7.2, the data from our trials will be analyzed if this proved to be the case and how future trials may be altered to improve the efficacy of an AR setup.

## 4. Experimental Setup

### 4.1. Phantom Parameters

The tumors embedded within the phantom were designed to model real tumor sizes. Tumor sizes of 30, 20, and 10 mm relate to a T2 tumor (tumor > 20 mm but ≤ 50 mm in greatest dimension), T1c tumor (tumor > 10 mm but ≤ 20 mm in greatest dimension) and T1b tumor (tumor > 5 mm but ≤ 10 mm in greatest dimension) (Edge et al., 2010). The tumors were modeled as perfectly round spheres. Both the phantom and the tumor were created using super soft plastic (M-F Manufacturing Company, Fort Worth, Texas, USA) to simulate the characteristics of human tissue. The tumors embedded within the phantom have the same material properties as its surroundings, which replicate the behavior of a non-palpable tumor.

**Figure 4:**
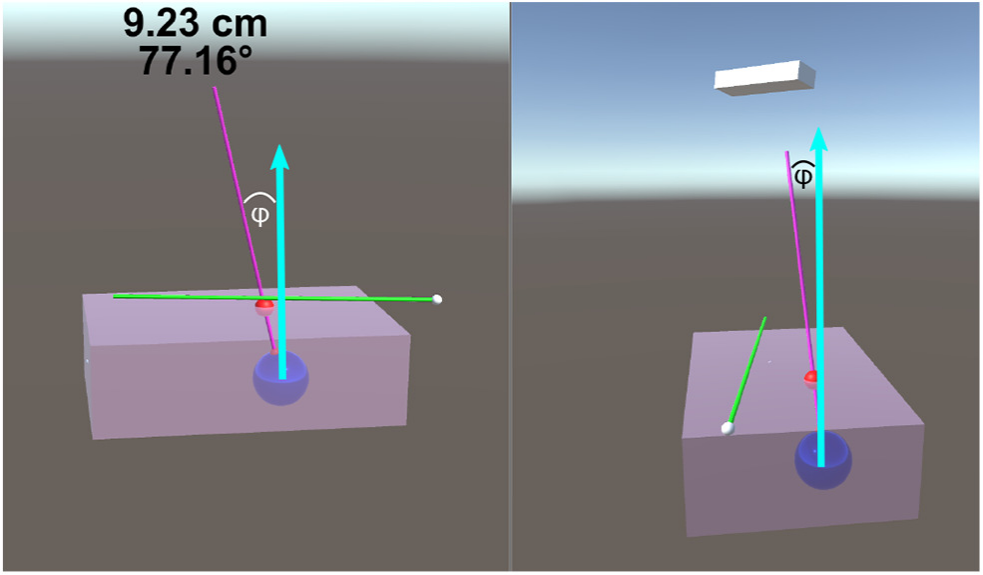
This image is a screenshot of what is displayed on the TV setup. The blue arrows in the figure were added in post-processing to clarify the measured value of ϕ. The left display shows the virtual scene from the perspective of the participant. The right screen shows a side view (from the right side of the table in Figure 1B). On the top of the left display are two numbers that are continuously updated. The number in the first row represents the Euclidean distance of the desired needle tip position compared to the actual tracked needle tip position. The number in the second row represents the angle difference of the ideal and actual needle represented in degrees. The angle between the normal vector and the ideal needle position, ϕ, was modulated between 0 and 40°.

The size of the phantom brick is 100 ×150 ×45 mm. The center of the tumor spheres were 40 ± 2 mm from the top surface. This depth was chosen as the average depth of a tumor (for breast cancer patients) was found to be 48 ± 13 mm (Aghili et al., 2015). The insertion depth can be modulated by changing the insertion angle of the needle, ϕ, as seen in Figure 4. Throughout the experiments, the angle between the ideal trajectory and normal vector was modulated between 0 and 40°. The rotation around the normal axis was chosen to be between 0 and 180°. The material used to create the phantoms was M-F Super Soft Plastic and was chosen to replicate the material properties of soft tissue.

### 4.2. Electromagnetic Tracking

Electromagnetic tracking involves the use of two systems: a magnetic field generator (source) and a magnetic sensor (tracking device). These systems use Faraday's law in which a field generator produces a known varying magnetic field, which induces a current in the tracking device. By measuring the current induced in the tracker, the position and orientation of the tracker can be obtained with sub-millimeter accuracy in ideal conditions (Lugez et al., 2015). These types of trackers are ideal for surgical settings due to their small sensor size and ability to track without a clear line of sight.

The device used for the experiment outlined in this paper was the NDI Aurora V2 System (NDI Medical, Waterloo, Ontario, Canada), which includes the field generator (shown in Figure 1A) coupled with three 6-DOF sensors (item ID 610029, shown in Figure 2B). These trackers were used to track the position and orientation of the needles and tumors.

As it was not practical to insert the sensor into the biopsy needle, a coordinate transform was calculated to map the trackers' position and orientation to the needle tips position and orientation. This calibration was done through an application named 3D Slicer (Fedorov et al., 2012) using the SlicerIGT module (Ungi et al., 2016). The root-mean-square error was found to be 0.03, indicating that the transform accurately maps the tracker to the needle tip (assuming minimal needle deformation).

### 4.3. System Development

Initially, the only information displayed to the first three participants was a model of the: tracked biopsy needle, the tracked ideal needle position and orientation, the tumor to be biopsied, and in the case of the TV setup, a transparent phantom. The system was augmented to display the Euclidean distance of the tip and the angle offset of the ideal needle trajectory and the tracked biopsy needle, which significantly increased user performance. Additionally, a large red ball was placed on the ideal needle trajectory showing the participants where the insertion point is located on the phantom. These changes can be seen in Figures 3, 4. It should be noted the three initial users data was removed from the results and analyses of this paper as substantial changes were made to the experimental setup after their trials.

### 4.4. User Sample Size

The sample size of an experiment is an important factor that can add validity to results obtained from user trials. For this study, 30 samples per visualization method were gathered. This number was chosen as the number of samples obtained from Kettenbach et al. (2005) was 20. A buffer of 1.5 times was applied to this study to account for differences in experimental setups. As each participant creates three samples per visualization method (one for each size of the tumor), we decided to recruit 10 volunteers for this experiment. As the initial trials suggested that each trial would take 1 h per participant, a 150% increase from previous studies seemed appropriate.

## 5. Visualization Setups

The visualization setups were the primary platform used to relay information to the participants. For both setups proposed in this paper (AR and TV), the Unity Engine was used to develop the virtual environments. Information from the NDI Aurora trackers was streamed to Unity through the PLUS server (Lasso et al., 2014). For the AR display only, head position data was also streamed to the Unity Engine through a C# program. As Unity operates in a left-hand coordinate system and the rest of the streamed data used the right-hand coordinate system, several C# scripts were developed within Unity to transform the data to one unified coordinate system. It should be noted that the display seen by the participant (as shown in Figures 3, 4) does not match what would typically be seen through an x-ray, MRI, or US image. As the purpose of this experiment was to compare the targeting accuracy of two different imaging modalities, it is assumed that a working model of the tumor has been created (either through x-ray, MRI, or US images) and that this model is registered to intra-operative scans (briefly described in section 2.2).

**Figure 5:**
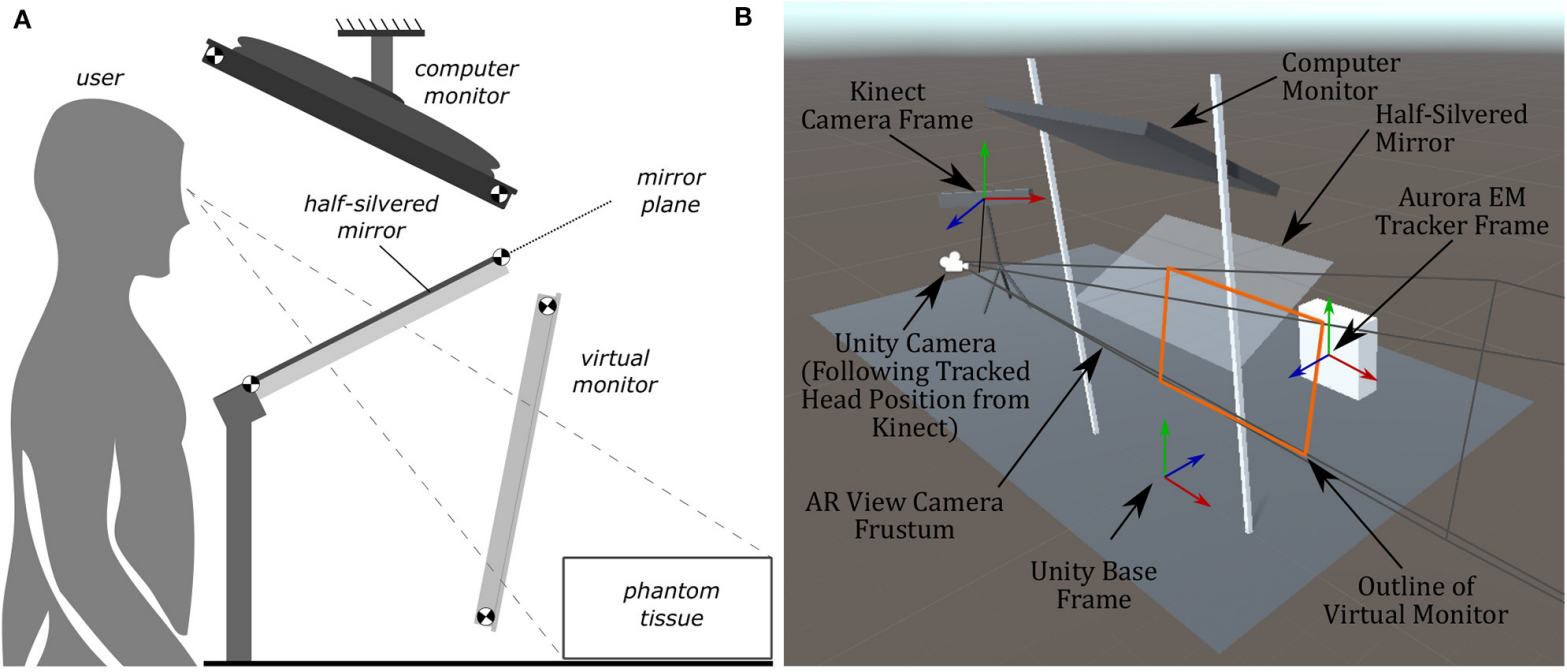
Diagrams of the AR display, showing the computer monitor, half-silvered mirror, and the location of the virtual monitor seen by the user. The virtual monitor is not a physical entity but is a reflected version of the computer monitor. The image of the Unity model shows coordinate frames of the EM tracker, Kinect, and camera position. **(A)** A side view illustration of the AR display used in the experiments. **(B)** The AR display modeled within Unity used to create the rendered image presented to the user.

To ensure the virtual scene matches the behavior of the real world, several steps have to be taken. In the Unity scene, game objects were created to represent physical objects in the experiment. Some game objects created for this experiment include the: Kinect camera, head position, semi-transparent mirror, monitor, phantom, and biopsy needles. The dimensions of these objects were measured, and 3D models were created to represent these objects in the Unity scene. These objects were placed in the virtual scene as they appeared in the experimental setup (see Figure 5B). To accomplish this, a base frame was created that was positioned directly in-between the two metal uprights of the AR setup and directly overtop the wooden board (see Figure 1A). The displacement of these objects in the experimental setup were measured from the base frame (x, y, z positions), and those displacements were implemented in the Unity scene. For rectangular objects, including the semi-transparent mirror and monitor, four position vectors were measured (representing the corners of the rectangle), and these values were implemented into Unity. For objects like the Kinect however, only the center position of the 3D camera was measured, and the angular offsets were first approximated, then finely tuned to match its orientation in the real world. All tracking information, measured in the base frames of the EM tracker and Kinect camera, respectively, was transformed by a rigid registration to the Unity base frame for use in the visualization technologies.

### 5.1. TV Visualization

The first system developed in this paper was the TV visualization setup which provided the user with two 2D projections of the 3D scene. The virtual environment in Figure 4 correlated with the physical system as the tumor and needles were tracked and updated in real-time. This means that each participant was able to receive tactile feedback from the phantom when the virtual needle was inserted into the virtual phantom.

Providing the user with two different scene views simultaneously offers a substantial benefit compared to a single view as depth data is often hard to perceive in a 3-D image projected onto a 2-D display. It should be noted that some users in our user trials provided feedback stating having two screens was dis-orientating; however, given enough training on the system, it is believed that this would overall benefit the end-user.

In a similar manner to the TV display, the goal of the AR display is to provide the clinician (or user) with information about the desired needle target location and angle of insertion. The AR display (see Figure 1A) is designed as a reach-in system, where a computer monitor is suspended above a half-silvered mirror (Bimber, 2002), and is an advanced version of our previous AR prototype (Rossa et al., 2018). As the user looks through the half-silvered mirror, they see both the image displayed on the computer monitor and the surgical scene (including the physical tumor phantom and biopsy needle) during the biopsy procedure. The images displayed on the computer monitor will appear to float in space behind the mirror and in front of the surgical scene. Therefore, the AR setup allows for an x-ray vision like visualization of the desired needle trajectory and tumor location to be presented to the user on top of the physical tumor phantom.

For the information to be displayed to the user, the same virtual environment used for the TV visualization (within the Unity game engine) will be used. The AR display provides a monoscopic, or single-camera projection, view of this virtual environment which is rendered in real-time in such a way that the overlay of the virtual tumor will match the position and orientation of the physical phantom tumor. Using the Kinect head-tracking data, the position of the camera (for rendering the virtual environment) is updated in real-time to match a center position between the user's eyes as they move. This live updating of the projection of the virtual environment provides an immersive display for the user, where matching the projection of the virtual environment to the user's vantage point will provide an impression of depth to the rendered image. This technique is known as motion parallax and provides sufficient visual cues for the user to perceive the full 3D structure of the virtual environment, as if a stereoscopic view of the virtual environment was being provided to the user (Howard, 2012). For this work, a fixed offset is used to transfer the head position tracked by the Kinect to determine the center position of the user's eyes (and therefore Unity camera position). While this fixed offset methodology was sufficient for this work, due to the simple geometric models being projected, tracking the user's eyes directly and calculating the center position may be advantageous in future work requiring higher visual fidelity.

To achieve the motion parallax effect, the physical layout of the AR setup will be examined to find the projective parameters of the rendering camera within Unity. Being as the computer monitor for the AR setup is reflected by the half-silvered mirror, a virtual monitor floating in space from the user's point-of-view will be considered. The location and orientation of this virtual monitor can be found through analysis of the physical layout of the AR setup. To do this, points at the four corners of the screen of the computer monitor in space, denoted as ${\;}^{s}P_{i}$, and the four corners of the half-silvered mirror, denoted as ${\;}^{m}P_{i}$, are used (where {*i* ∈ ℕ|1 ≤ *i* ≤ 4}). These corner points can be measured directly or can be calculated using the height at which the computer monitor and the mirror are placed and the angles of the computer monitor and mirror relative to the desk surface. From the set of points ${\;}^{s}P_{i}$, the normal vector ${\;}^{s}\vec{n}$ for the computer monitor can be found and, in the same manner, the normal vector for the mirror ${\;}^{m}\vec{n}$ can be found from the set of point ${\;}^{m}P_{i}$. Using the normal vector ${\;}^{m}\vec{n}$ of the mirror, the shortest line $\vec{\ell_{i}}\left(t\right)$ between the plane of the mirror and each point ${\;}^{m}P_{i}$ can be found such that

(3) $$\begin{array}{l} \vec{\ell_{i}}\left(t\right){=}^{m}P_{i}{+}^{m}\vec{n}t \end{array}$$

where *t* is the parametric variable of the line (*t* ∈ ℝ). As with the approach outlined in Rossa et al. (2018), the plane of the mirror is considered to be infinitely large and therefore we can solve for the value of the parametric variable *t* for each line $\vec{\ell_{i}}\left(t\right)$ at its intersection with the mirror, where ${\;}^{I}t_{i}$ is the value *t* at the point of intersection and the point of intersection is PI i=ℓ→i(tI i). The reflected virtual monitor points ${\;}^{v}P_{i}$ are given by

(4) $$\begin{array}{l} {\;}^{v}P_{i}{=}^{I}t_{i}{+}^{m}{\vec{n}}^{I}t_{i} \end{array}$$

such that the reflected point ${\;}^{v}P_{i}$ is the same distance away from the mirror as its corresponding monitor point ${\;}^{s}P_{i}$.

With the locations of the corners of the virtual monitor (${\;}^{v}P_{i}$) now known, the parameters of a generalized perspective projection (Kooima, 2009) for the rendering camera of the virtual environment can be found. Through the technique outlined in Kooima (2009), the generalized perspective projection is calculated using the location of the virtual monitor and the Unity camera position (equivalently a point between the user's two eyes). The resulting rendered image, after considering the AR layout and camera projection, can be thought of as treating the virtual monitor as if it were a window through which the user is looking. Figure 5B shows the Unity camera frustum resulting from the generalized perspective projection calculations, with the edges of the frustum going through each of the four corners of the outline of the virtual monitor. As the user moves their head, the rendered image is updated to match the user's point of view through this window, which therefore achieves the desired goal of matching the position and orientation of objects within the virtual environment with their respective physical counterparts.

## 6. Results and Analysis

Throughout the trials, several pieces of information were gathered, including both quantitative and qualitative data. Mean values for the quantitative data for the AR and TV setups can be found in Table 1. The data in these tables show the average insertion angle deviation (defined in section 3.1), Euclidean distance from desired end location to observed location (named Euclidean distance offset, defined in section 3.1), and the amount of time taken to perform each procedure for each individual user. It should be noted that the average values do not offer a full perspective on the results as learning appeared to be a factor among trial; however, this is discussed in section 7.2.

Each trial focused on localizing the needle to a specific point on an artificial tumor within the phantom. Although the size of the tumor changed between trials, the task itself stayed relatively consistent within the AR and TV trials. The localization task was very similar within each visualization method meaning the difference in difficulty when guiding the needle to a 10 mm target compared to a 30 mm target was negligible. Although there is no increased difficulty within each visualization modality, there is a possibility of learning occurring throughout each trial. As each participant has never used this system before, there is the possibility that their performance could improve in the final localization exercise due to improved familiarity with the system.

For the above reasons, the trials were analyzed on a per-trial and per-setup basis. The per-trial data had 10 data points per trial, as 10 participants were performing each trial once (60 total). Combining the data for each setup creates 30 data points per visualization display (60 total). Additionally, analysis of each participant's data was performed to add further perspective in the discussion (section 7).

Within the data, some trials were found to be outliers. Removing the outlier trials from the data, as defined by a value that is more than three scaled median absolute deviations away from the median, cleaner results can be found. As the users were told not to worry about time, the outliers' analysis depended only on the angle and position data. If any participants' trial had an outlier in either the angle or position value, the data for that trial was removed from the cleaned data. After removing the outlier data, there were 25 data samples for the AR setup and 29 for the TV setup (54 total).

### 6.1. Position Error

**Figure 6:**
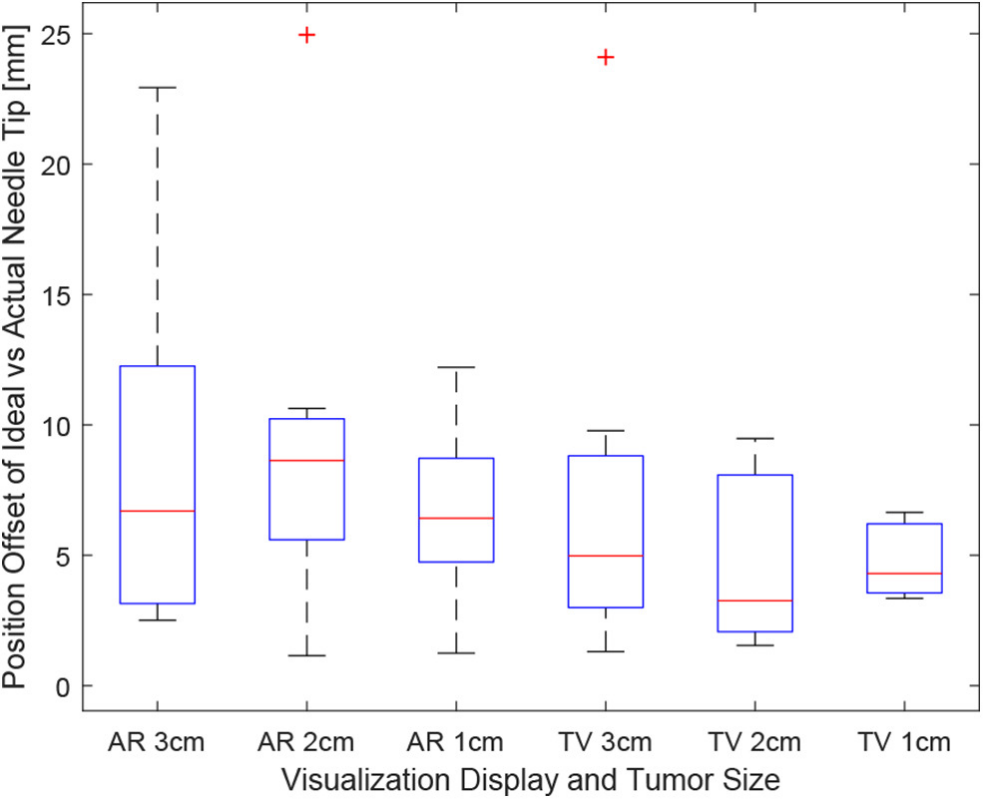
This image shows the box-plot of the position offset of the needle tip for each user trial. The position offset is calculated using the Euclidean distance between the desired and actual needle tip position (measured in millimeters). The data of all 10 participants were combined to create this box-plot. The red crosses represent outliers in the data.

**Figure 7:**
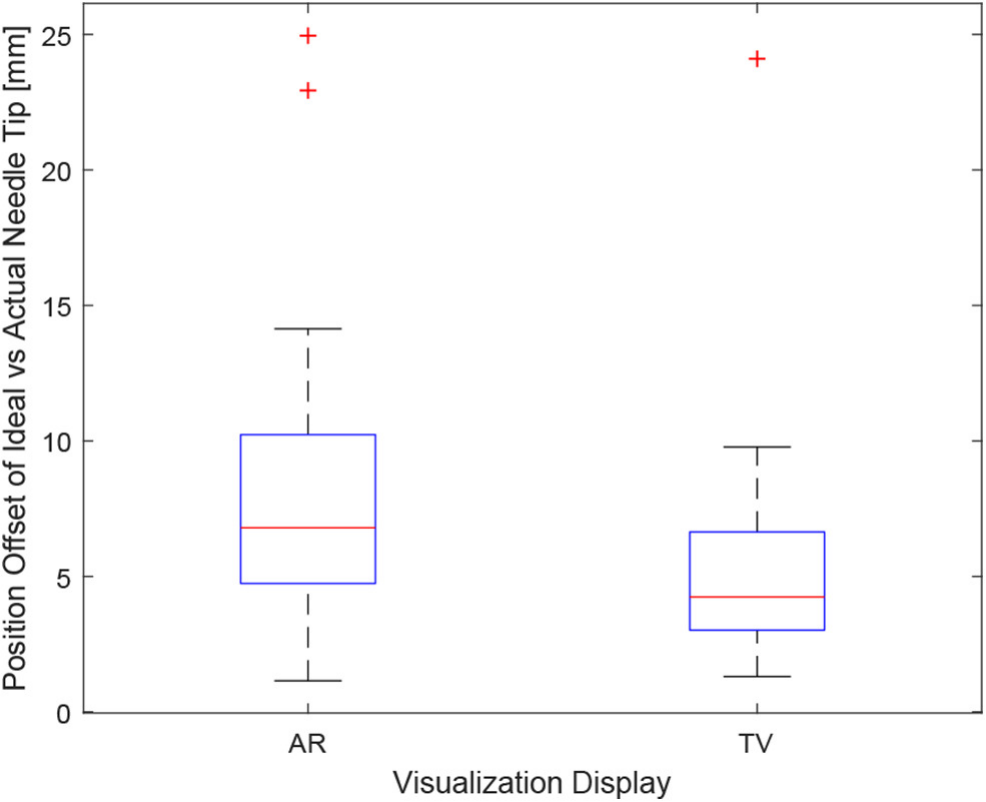
This image shows the box-plot of the position offset of the needle tip for the AR setup and TV setup. The position offset is calculated using the Euclidean distance between the desired and actual needle tip position (measured in millimeters). The data of all 10 participants were combined for the 30, 20, and 10 mm trials to create this box-plot. The red crosses represent outliers in the data.

Positioning is an important aspect of biopsying as imprecise positioning may lead to the desired tissue sample not being extracted. The box-plot shown in Figure 6 depicts all the participants' data combined for each of the 6 trials. Taking a courser look at the data, Figure 7 shows the position data when all the AR and TV trials are combined. Removing the outlier data from the positioning data, the new per-setup mean was found to be 6.48 ± 3.21 (mm) for the AR display and 4.87 ± 2.52 (mm) for the TV display. Paired *t*-test data showed that both the AR and TV display had equal means for the Euclidean distance offset (null hypothesis was accepted); however, the power of the test was found to be 0.41. Further analysis of these values are discussed in section 7.2.

**Figure 8:**
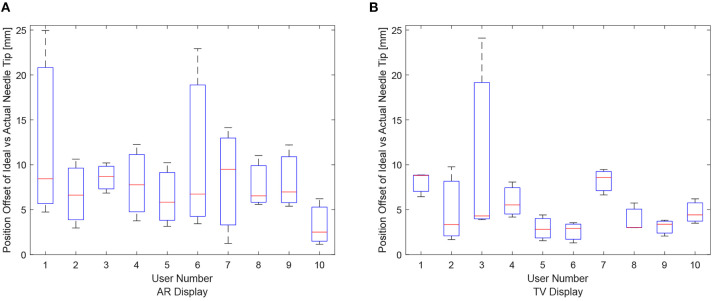
The needle tip position offsets for each user for the AR and TV displays. The position offset is calculated using the Euclidean distance between the desired and actual needle tip position (measured in millimeters). These plots show the data from the 30, 20, and 10 mm trials combined per participant. **(A)** The needle tip position offset for the AR display. **(B)** The needle tip position offset for the TV display.

The data for each users positioning performance given the virtualization display is shown in Figures 8A,B. The variance was calculated for each user using either the AR or TV display. All the users' data variance data were pooled together, and with the outliers removed, it was found that the mean variance for the AR display and TV display was 10.91 and 2.07°, respectively. From the mean variance results. It can be seen that users typically had greater precision when using the TV display compared to the AR display, which was further confirmed from a paired *t*-test. It should be noted that more trials will need to be performed to confirm this hypothesis (144, according to calculations) as the current power of the hypothesis test is 0.09.

### 6.2. Angle Error

**Figure 9:**
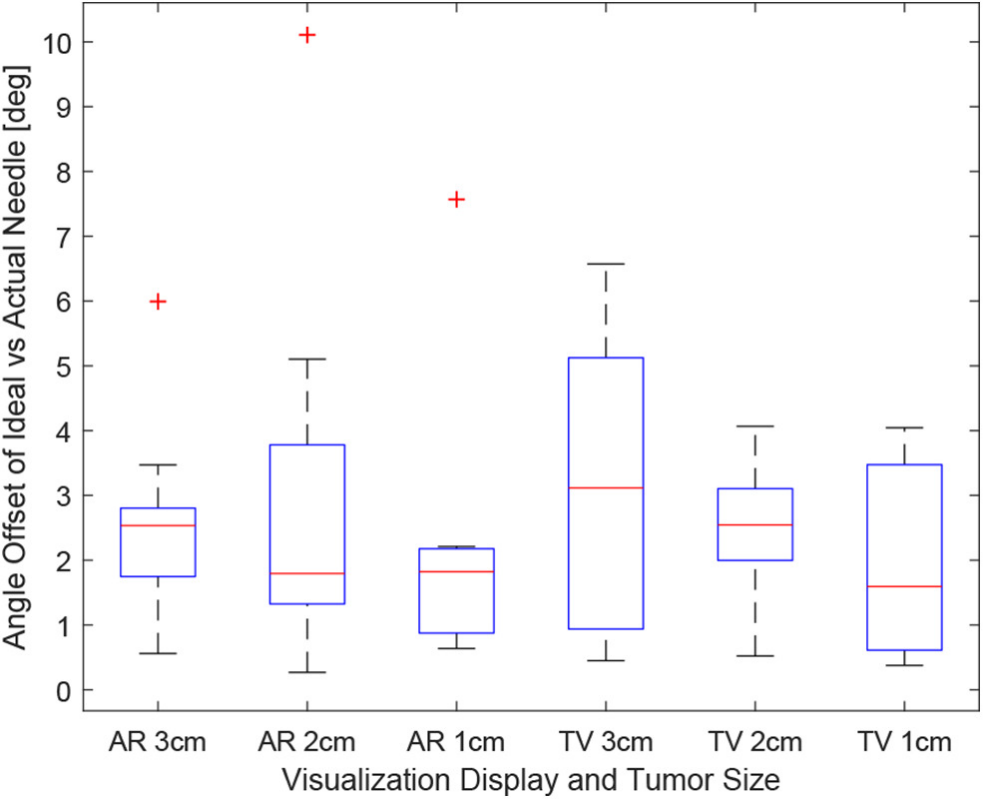
This image shows the box-plot of the angle offset of the needles for each user trial. The angle offset is defined by the difference in angle between the ideal trajectory vector and the actual needles vector (measured in degrees). The data of all 10 participants were combined to create this box-plot. The red crosses represent outliers in the data.

**Figure 10:**
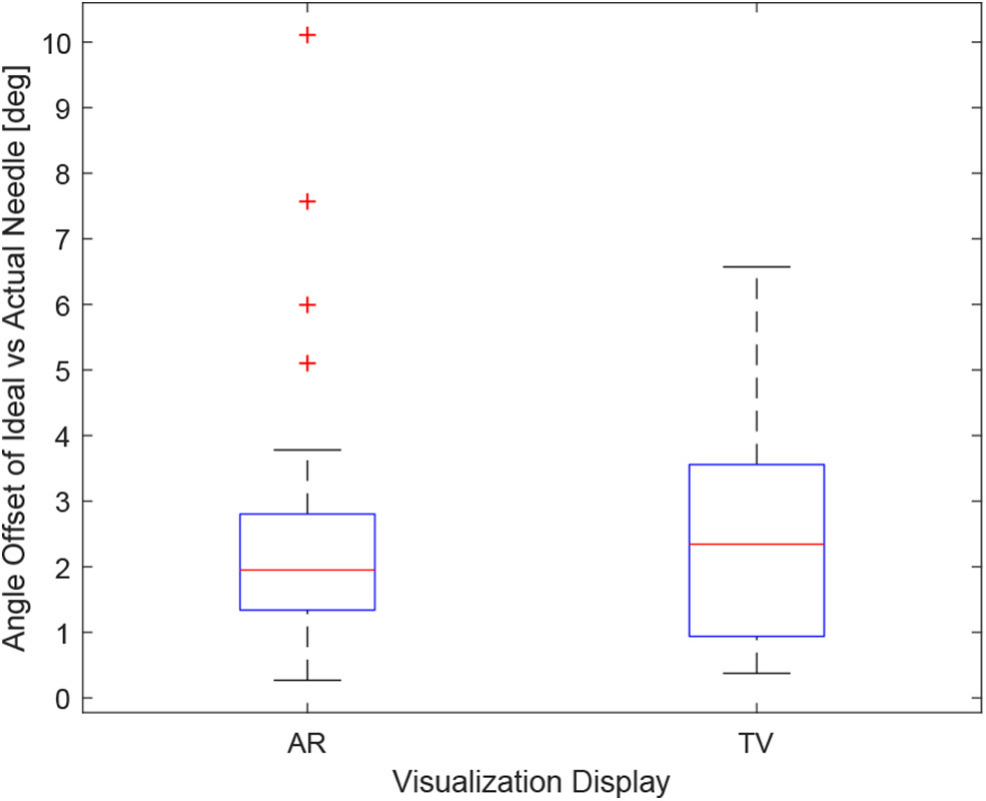
This image shows the box-plot of the angle offset of the needles for the AR setup and TV setup. The angle offset is defined by the difference in angle between the ideal trajectory vector and the actual needles vector (measured in degrees). The data of all 10 participants were combined for the 30, 20, and 10 mm trials to create this box-plot. The red crosses represent outliers in the data.

Obtaining the proper angle of insertion during a biopsy procedure can be important as it can lead to better positioning and helps avoid delicate tissue within the body. Looking at the results in Figure 9, it can be seen that the angle differences between the AR and TV setup are minimal [considering that the RMS accuracy for trackers is 1.3° (Lugez et al., 2015)]. Looking at the data as a whole in Figure 10, it can be seen that the AR display may provide more precise angle positioning (when removing the outliers). When the outliers of the data were removed, the new mean was found to be 1.97 ± 1.08° for the AR display and 2.61 ± 1.66° for the TV display. Paired *t*-test data showed that both the AR and TV display had equal means for the angle offset (null hypothesis was accepted); however, the power of the test was found to be 0.53. Further analysis of these values are discussed in section 7.2.

**Figure 11:**
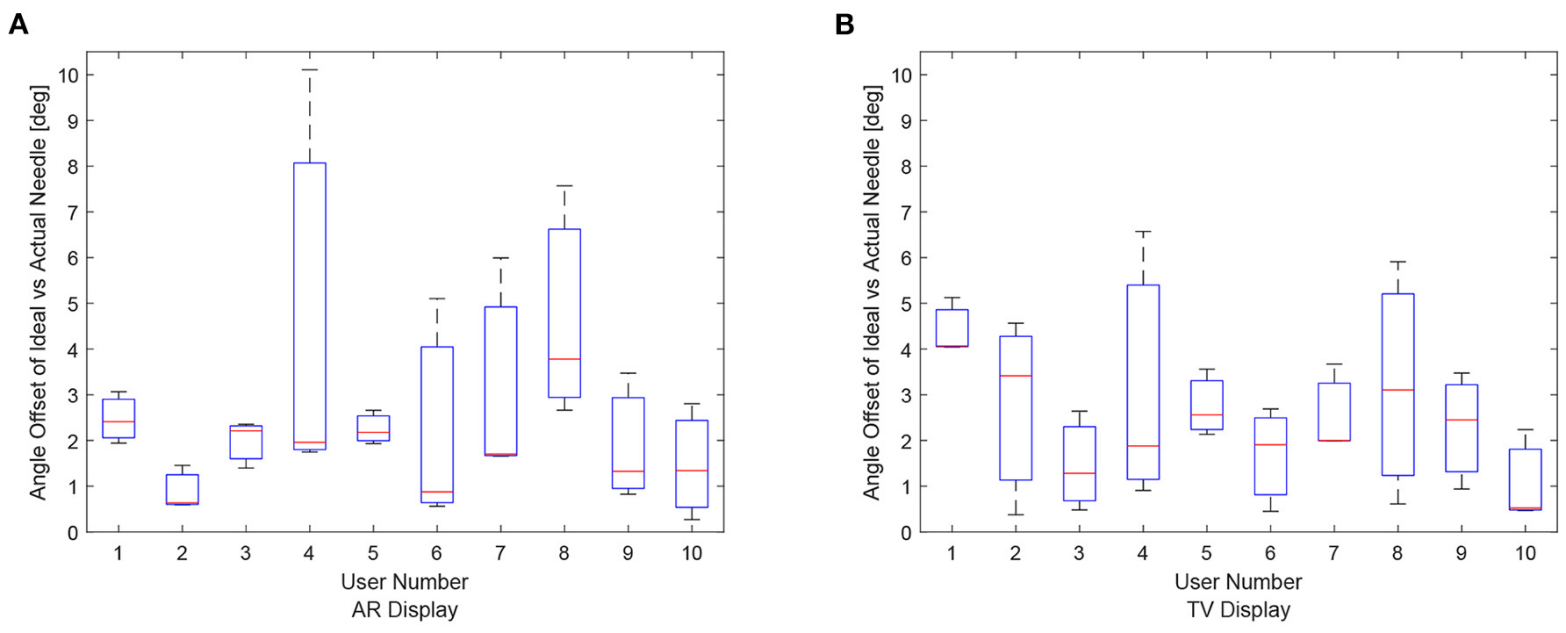
Angle offset of the needle tip for each user using the AR/TV setup. The angle offset is defined by the difference in angle between the ideal trajectory vector and the actual needles vector (measured in degrees). The data from the 30, 20, and 10 mm trials were combined per participant to create these box-plots. **(A)** This image shows the box-plots of the angle offset of the needles for each user using the AR setup. **(B)** This image shows the box-plots of the angle offset of the needles for each user using the TV setup.

The data for each users angle positioning performance given the virtualization display is shown in Figures 11A,B. The variation between users seems to be greater in the AR setup compared to the TV setup.

### 6.3. Procedure Time

**Figure 12:**
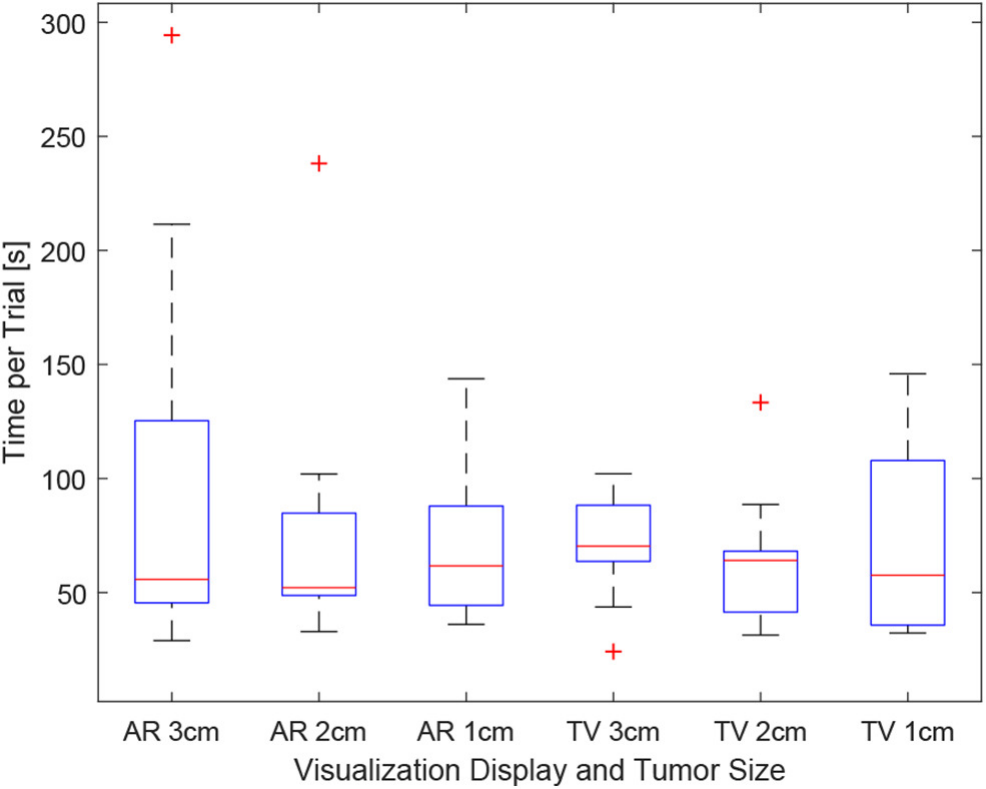
This image shows the box-plot of the time taken to complete each user trial. The data of all 10 participants were combined to create this box-plot. The red crosses represent outliers in the data.

**Figure 13:**
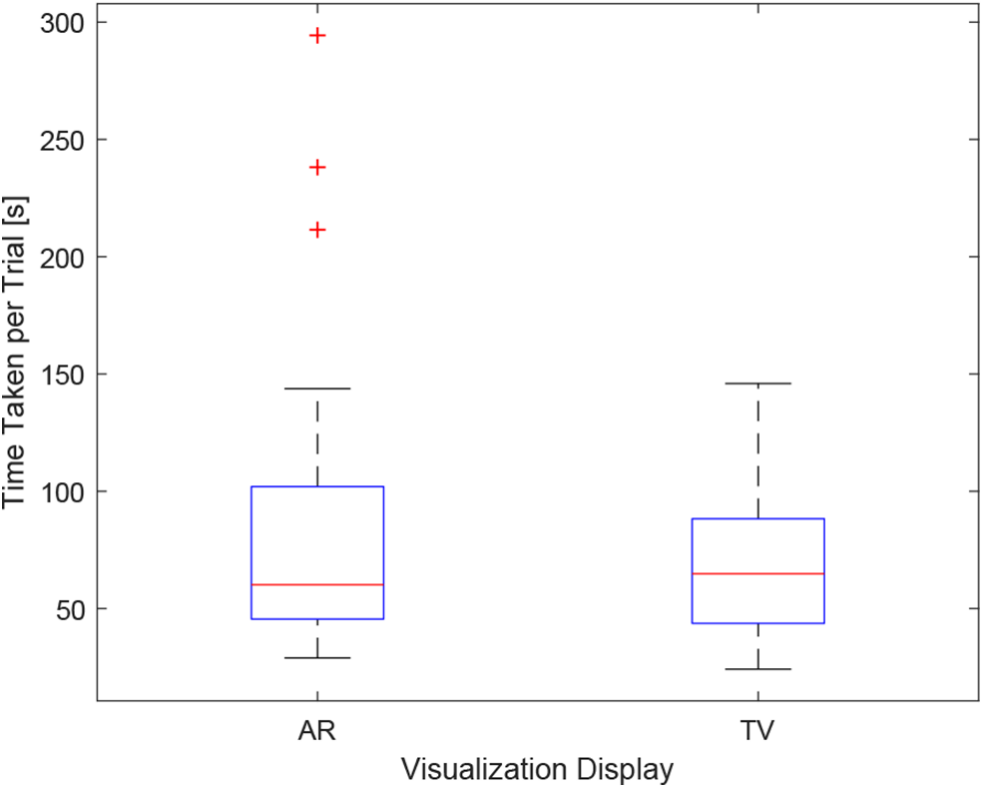
This image shows the box-plot of the time taken to complete each user trial with all the AR and TV trials grouped into one category. The data of all 10 participants were combined to create this box-plot. The red crosses represent outliers in the data.

As the users were not instructed to minimize their procedure time, the time reported indicates the time taken to accurately position the needle to the best of the participants' ability. It can be seen in Figure 12, the times were relatively consistent regardless of the display used and the size of the tumor. This point is further expressed in Figure 13 where the box-plots look very similar (without the outliers). After removing the outliers from the data, the new mean values were 82.75 ± 61.76 (s) for the AR display and 68.27 ± 32.29 (s) for the TV display. The power of the paired *t*-test was found to be 0.13, which renders any paired *t*-test results not statistically significant.

### 6.4. Qualitative Data

The questionnaire included both multiple-choice data and short answer data. The multiple-choice responses can be seen in Table 2, while the short answer questions can be found in section 7.1. The numbers in Table 2 for the *Task Difficulty* section represents whether the user felt the tasks were easy (1) or hard (5) using that specific setup. A similar 5 point scale was used for the *Confidence in Positioning* section where a value of 1 represented the user felt they were very accurate using the specific setup, and a value of 5 indicates that the user felt they were very inaccurate in their positioning. The wording used in the questionnaire (*Task Difficulty* and *Confidence in Positioning*) was the exact wording used in the questionnaire. A category for system preference was also included in the questionnaire. It should be noted that long answer responses to perceived advantages and disadvantages for each system were also gathered from the questionnaire for each user.

**Table 2:** Questionnaire results.

	**AR display**		**TV display**		
**Participant number**	**Task difficulty**	**Confidence in positioning**	**Task difficulty**	**Confidence in positioning**	**System preference**
1	3	3	2	2	TV
2	3	3	3	3	Neither
3	2	2	4	2	AR
4	5	5	3	1	TV
5	2	2	2	2	Neither
6	2	2	3	2	AR
7	3	3	2	4	TV
8	4	4	3	3	TV
9	2	2	3	3	AR
10	3	3	4	4	TV

The wording used in the questionnaire for the long answer responses were *Perceived advantages of AR/TV* and *Perceived disadvantages of AR/TV*. The user-perceived task difficulty score for the AR setup was found to be 2.90 ± 0.99 compared to the TV setup in which the score was 2.90 ± 0.74. The confidence in the positioning score for the AR setup was 2.80 ± 0.63 compared to the TV setup score of 2.60 ± 0.97.

## 7. Discussion

The introduction of sensorized surgical equipment, coupled with proper ways to relay sensor information in a meaningful way to physicians, can dramatically improve the accuracy and time of surgical procedures. Although historically, head-mounted devices lacked characteristics to make them suitable in the operating room (field of view, resolution, size) (Keller and Colucci, 1998), new technologies like the Hololens and Google Glass have found popularity in surgical settings (Iqbal et al., 2016; Sauer et al., 2017). There are limitations with these head-mounted devices, including their field of view, tracking capabilities, and resolution (1268 × 270 per eye for the Hololens). However, in a future study, we hope to implement AR using a head-mounted device to see if biopsying procedures can be done accurately through these devices.

### 7.1. User Reviews

As this project was intended to aid physicians in needle-guided surgeries, specifically biopsies, it is essential to gain feedback from the participants in the study. At the end of each trial, the users submitted individual testimonials for the perceived advantages and disadvantages for each setup (resulting in four short answer responses from each participant). For the AR setup, many users felt that relaying the information directly over the work-space was intuitive and convenient while they performed the procedures. However, in the current setup, the primary concern was a single display could not provide sufficient depth data (even with the head racking).

For the TV trials, users felt that having two displays was invaluable for needle targeting accuracy. Some users reported that having the front and side views allowed them to focus on live image data instead of relying heavily on the projected angle and distance numbers. Despite the advantages provided to some users from the secondary monitor setup relating to positioning accuracy, it appeared that many participants found having the secondary point of view was initially very confusing.

The AR display drew more varied responses from the participants. For some participants, having the objects displayed directly over their corresponding physical entities were more intuitive; however, others often had trouble getting used to the setup. In this study, only a single view was provided in the AR display, which proved problematic as some participants had trouble minimizing the angle offset values during insertion. Although the head tracking provided a stereoscopic view of the virtual environment, users often didn't move their head much during the trials, reducing the amount of depth data shown to them. The user trials for this experiment involved a static positioning task coupled with a fixed-base AR display. As static targeting tasks don't require much movement from the physician, it is felt that the capabilities of an AR setup weren't fully utilized. In future work, a head-mounted display like the Hololens could be used, coupled with a dynamic targeting task, to determine if such an AR setup offers a significant advantage compared to a second screen visualization similar to the one outlined in this paper.

From the user reviews, it is clear that having both a front and side view of the surgical scene is essential for perceived accuracy. In future studies, the AR display will be augmented to include a side profile view to portray the depth data to the physician/user efficiently. This display could be shown on the corner of the display, or the user could toggle between views using a foot pedal or other toggle switch. Despite the initial confusion of the two displays, offering the secondary point of view in the AR setup should give physicians more confidence in their positioning and should make the task less difficult with training.

### 7.2. Interpreting Results

The results in sections 6.1–6.3 provide promising values; however, a more in-depth analysis of these values is required to interpret the values correctly. As data was only collected from 10 users, with some of their trials being removed through an outlier analysis, a power analysis was performed to determine the power of a two-sampled *t*-test. For each variable (Euclidean difference, angle offset, time), the null hypothesis is defined as both systems (AR and TV) having equal means and variances in terms of any criterion. The *t*-test determines whether the null hypothesis is accepted or rejected; however, the power determines how valid the *t*-test is given the amount of data provided.

The power score for the Euclidean difference values, angle offset values, and time for the TV setup vs. the AR setup was found to be 0.41, 0.53, and 0.13, respectively. These results are statistically low compared to the more accepted value of 0.8–0.95 (Whitley and Ball, 2002). From these results, it is clear that the calculation for the sample size in section 4.4 was not suitable for this application. Despite the low power score, analysis of the results can still be conducted, keeping in mind that there is a higher possibility of an accepted null hypothesis being incorrect (false positive). Furthermore, appropriate sample sizes will be stated to ensure researchers are able to choose adequate sample sizes in the future (given similar trial conditions).

Through further analysis of the Euclidean difference power score, it was found that ~64 trial data points are needed to obtain a power value of 0.8. As three samples were collected per participant, a minimum of 22 participants should be recruited to add more validity to future studies. Performing a two-sampled *t*-test on the results showed that the null hypothesis was accepted with a 97.76% confidence. From the data collected, it appears that there is no discernible difference in performance for the needle tip positioning; however, more trials will need to be conducted to verify that this result is accurate.

Looking at Figure 6, it appears that the first AR trial had the most significant variance in positioning precision. For many participants, this was their first time using an AR setup, which proved to be very difficult to get used to during the short duration of each trial. As the trials progressed to the smaller tumor sizes, it appears as though the participants were able to locate their needle close to the tumors more precisely. In the future, more practice time should be allotted to participants to familiarize them with the equipment used in the setup.

Analyzing the angle offset values between the two virtualization setups, a suitable number of samples to improve the power of the paired *t*-test to 0.8 was found to be 47. Translating that number to a participant number, at least 16 participants need to be recruited. From the paired *t*-test, it was found that the null hypothesis was accepted once again. This further leads to the idea that the two systems provide similar outcomes.

Inspecting the angle values further, it appears that they are all quite low (1.97 ± 1.08 and 2.61 ± 1.66° for the AR and TV values, respectively). As the RMS accuracy of the tracking devices was found to be 1.3° (Lugez et al., 2015), the extracted angle values were incredibly accurate for manual performance.

As the participants were not instructed to minimize the time per procedure, it is understandable that the power score for the time analysis was low (0.13). Each participant had varying levels of skill coming into the trials, and the time varied significantly between participants (see Table 1). As this power level is very small, any paired *t*-test would not yield meaningful results.

### 7.3. Participant Sampling

In this experiment, the individuals who participated in this study did not have any previous medical experience. The background of the participants includes graduate students, nursing students, and engineers. Choosing a novice set of individuals yielded both positive and negative results. On one side, the results obtained from the experiment were positive as novice individuals were able to achieve sub-centimeter and sub-degree precision in a procedure entirely new for them. On the other hand, the variability between participants, and even among participants but between trials, yielded low power results in the statistical analysis. In this study, it has been proven that using these augmented visualization methods, users can perform a simple biopsy with adequate precision. However, it has yet to be proven that these modalities would offer a significant benefit to a clinician. In future experiments, the participants chosen should have proficiency in biopsy procedures. This change would more effectively test as to whether this is a worthwhile technology to bring into the greater medical field. Additionally, medical professionals may have more familiarity working with augmented reality system, and the variance among trials may be decreased.

### 7.4. Other Applications

From the analysis, it was found that an AR setup is non-inferior to a TV setup. This paper focused on biopsying, a procedure that primarily targets static points; however, AR has an excellent chance to perform better for dynamic trajectory following and dynamic target acquisition. One area in which an AR display could be useful is beating heart surgery. The development of 3D ultrasound imaging could enable surgeons to perform minimally invasive surgeries on beating hearts, and technologies have been developed to increase the performance of the surgeons (Yuen et al., 2009; Cheng and Tavakoli, 2018). Further research could reveal that projecting the visual data over the patient, using an AR setup similar to that proposed in this paper could increase the performance of the surgeons.

## 8. Conclusion

This work aims to provide physicians with an alternative method to visualize ideal and actual needle trajectories for their biopsy needles in an intuitive and impactful way. As shown in this paper, this system has allowed inexperienced users the ability to localize needles given complex angles with sub-centimeter and sub-degree precision.

In this paper, a virtual environment was created to display information relating to the tracked needle location, ideal needle trajectory and end position, and the tumor to be biopsied. The methods of presenting this information to the users were split into two distinct visualization methods. The first visualization technology was an artificial reality (AR) display that relayed the information directly over phantom and needle. The second method was a virtual environment that simulated the physical environment from both a front and side view. From the data obtained from the user trials, it was found that the users were able to achieve a Euclidean distance between the ideal end position of the needle and the actual needle tip of 6.48 ± 3.21 (mm) for the AR display and 4.87 ± 2.52 (mm) for the TV display. The angle difference between the ideal needle trajectory was found to be 1.97 ± 1.08° for the AR setup and 2.61 ± 1.66° for the TV setup.

Valuable data was extracted from the trials. From the users' subjective experience, it was found that providing two different planar views of the virtual scene improved user confidence. These views can easily be added to our AR display, which should improve the performance of the AR system. Objectively it was found that an AR setup is non-inferior to a TV setup for a static targeting task. Further research will have to be conducted to see if this is also true for more dynamic targeting tasks. In future works, we hope to make the user interface more intuitive to the user, and to transfer the AR display to a more portable device.

## Data Availability Statement

The datasets generated for this study are available on request to the corresponding author.

## Ethics Statement

The studies involving human participants were reviewed and approved by University of Alberta Ethics Board (Approval Number Pro00070096). The patients/participants provided their written informed consent to participate in this study.

## Author Contributions

DA-D and JC have done most of the technical work. The principal supervisor was MT. All authors contributed to the article and approved the submitted version.

## Conflict of Interest

The authors declare that the research was conducted in the absence of any commercial or financial relationships that could be construed as a potential conflict of interest.
